# Relationship between albumin-corrected anion gap and non-alcoholic fatty liver disease varied in different waist circumference groups: a cross-sectional study

**DOI:** 10.1186/s40001-024-01811-w

**Published:** 2024-03-27

**Authors:** Ye Lu, Ya-zhen Zhan, Wan Li, Shu-li Liao

**Affiliations:** Department of Gastroenterology, Shaoxing Central Hospital, No.1 Huayu Road, Keqiao District, Shaoxing, 312030 Zhejiang China

**Keywords:** Non-alcoholic fatty liver disease, Clinically significant fibrosis, Albumin-corrected anion gap, NHANES

## Abstract

**Objectives:**

To investigate the association of albumin-corrected anion gap (ACAG) with non-alcoholic fatty liver disease (NAFLD) and clinically significant fibrosis (CSF) defined by vibration-controlled transient elastography measurements.

**Methods:**

This cross-sectional study including 4531 participants was conducted using the data from the NHANES database of cycles 2017–2018. The outcomes were set as NAFLD vs. non-NAFLD and NAFLD with CSF vs. NAFLD without CSF. The generalized additive model and restricted cubic spline analyses were used to assess the nonlinear relationship. The generalized linear models, logistic regression models, sensitivity analysis, P trend test, subgroup analysis, and mediation analysis were employed to analyze the association. Finally, an ACAG-based model was constructed and evaluated.

**Results:**

A higher ACAG level was an independent risk factor for NAFLD (*P* < 0.05), but not for CSF (*P* > 0.05). The sensitivity analysis and *P* trend test results substantiated the significantly positive relationship between ACAG and NAFLD (*P* < 0.05). Interestingly, the obvious connection between ACAG and NAFLD varied in different waist circumference groups and played a central role in the central obesity group. In addition, alanine aminotransferase and waist circumference were the mediators in their relationship. Moreover, the ACAG-based model performed well in predicting NAFLD.

**Conclusions:**

ACAG level is independently associated with NAFLD but not CSF. ACAG might be a novel and reliable biomarker for predicting NAFLD clinically especially in the central obesity population.

**Supplementary Information:**

The online version contains supplementary material available at 10.1186/s40001-024-01811-w.

## Introduction

Non-alcoholic fatty liver disease (NAFLD) is defined as hepatic steatosis, which occurs in the absence of excessive alcohol intake, medications, or other specific causes of fatty liver disease [1]. The incidence of NALFD is 25.24% worldwide varying in different regions, with approximately 30% prevalence rate in the Middle East and South America countries [2]. There were 20–30% of adults and around 10% of children and adolescents who developed NAFLD in Western countries [3]. NAFLD is closely related to the presence of metabolic syndrome components such as adiposity, diabetes, dyslipidemia, and hypertension [4]. NAFLD may progress to hepatic fibrosis, cirrhosis, or hepatocellular carcinoma presumably resulting in the need for transplantation or even mortality [5]. Moreover, NAFLD has emerged as an additional, independent risk factor for atherosclerosis and cardiovascular diseases [6]. Hormonal changes are associated with NAFLD development and severity. Metabolic acidosis suppresses the levels of growth hormone and insulin-like growth factor-I, which are linked to an elevated probability of NAFLD prevalence and progression to cirrhosis or other more severe liver diseases [7]. Therefore, it is of great significance to identify an acid-related biomarker and concern its levels for preventing NAFLD escalation timely.

Anion gap (AG) is a commonly used indicator that reflects the concentration gradient between the positive and negative ions [8]. It is applied to assess the acid–base balance of blood fluids and classify the presence and severity of metabolic acidosis. Nevertheless, the albumin molecule carries a net negative charge and a change in its concentration can affect the AG results for patients with hypoproteinemia [9]. Therefore, the albumin-corrected anion gap (ACAG) was proposed to avoid false-negative results. ACAG upregulation had an intimate relationship with increased in-hospital mortality in patients with acute pancreatitis [10]. Zhong et al. observed a meaningful association of ACAG elevation at the initiation of continuous renal replacement therapy with intensive care unit mortality [11]. In addition, an increase in ACAG levels independently predicted in-hospital mortality among patients with heart failure [12]. However, the literature on the connection between ACAG and NAFLD is limited.

Herein, we conducted a cross-sectional study based on the National Health and Nutritional Examination Surveys (NHANES) (2017–2018 cycles) to determine the association of ACAG with NAFLD and CSF. More importantly, we investigated their relationship in different waist circumference groups to identify the target population. Finally, we conducted an ACAG-based model and examined its clinical usefulness among NAFLD patients.

## Materials and methods

### Study design and participants

We obtained the data from the NHANES (2017–2018 cycles), since the vibration-controlled transient elastography (VCTE) information is specifically included. Using a multistage and stratified sampling design, NHANES (https://www.cdc.gov/nchs/nhanes/index.htm) is a public database providing representative samples of the non-institutionalized U.S. resident population. The NHANES study protocol was approved by the Ethics Review Board of the National Center for Health Statistics Research (Protocol #2018–01) and all the participants provided signed informed consent. In this study, we retrieved publicly available data without personally identifiable information. The methods and results follow relevant National Center for Health Statistics regulations.

A total of 9254 samples were included in these two cycles. Exclusion criteria: missing VCTE data (*n* = 3306); elastography examination status was ineligible, not performed, or partial (*n* = 456); serologic positivity for viral hepatitis (*n* = 43); excessive alcohol intake defined as more than four or five standard drinks per day (*n* = 592); missing ACAG data (*n* = 326). Finally, we enrolled 4531 samples for analysis.

### Definition of NAFLD and clinically significant fibrosis (CSF)

The extra-large or medium probe equipped FibroScan^®^ model 502 V2 Touch (Echosens, Paris, France) was adopted to carry out the elastography examinations by an experienced technician in the NHANES mobile examination centers (MECs). A complete exam should meet the following conditions: fasting time of no less than 3 h, at least 10 complete measures, and the interquartile range/median of the liver stiffness measurement (LSM) < 30%. NAFLD was diagnosed according to the controlled attenuation parameter (CAP) value with a cutoff value of 285 dB/m [13] and CSF was defined based on the LSM value with a cutoff value of 8.6 kPa [14]. For sensitivity analysis, NAFLD was identified by setting CAP = 263 dB/m as the cutoff point [15].

The primary outcome: NAFLD *vs.* non-NAFLD. The secondary outcome: NAFLD with CSF *vs.* NAFLD without CSF.

### Variable extraction and ACAG calculation

Demographics: age, gender, waist circumference, poverty income ratio (PIR), smoking status (former smoker: smoked at least 100 cigarettes in life and not smoke cigarettes now; current smoker: smoked at least 100 cigarettes in life and still smoke cigarettes now; never smoker: not smoke at least 100 cigarettes in life) were collected from self-reported questionnaires in the MECs. Besides, hypertension and diabetes information were also obtained by the self-reported questionnaires. Serum biochemistry profiles: total bilirubin, total calcium, alanine aminotransferase (ALT), aspartate aminotransferase (AST), blood urea nitrogen (BUN), creatinine, glucose, triglyceride, high-density lipoprotein cholesterol (HDL-C), sodium, potassium, chloride, bicarbonate, and albumin were included. To avoid collinearity, sodium, potassium, chloride, bicarbonate, and albumin were only used for ACAG calculation, but were excluded in the statistical analyses.

AG was calculated according to the following equation: AG (mmol/l) = (sodium + potassium)—(chloride + bicarbonate). ACAG was determined using the formula: ACAG (mmol/l) = [4.4—albumin (g/dl)]*2.5 + AG [16].

### Statistical analysis

All data analyses were performed using the SPSS software (version 23.0) and R software (version 4.2.1). We summarized participant characteristics in the NAFLD and non-NAFLD groups, NAFLD with CSF and without CSF groups. Data were presented as count (percent) for categorical variables and the differences between the groups were compared by the Chi-square test. Since all the continuous variables did not conform to a normal distribution, they were expressed as median [interquartile range], and group differences were evaluated by the Mann–Whitney *U* test. The significant factors identified in this univariate analysis were selected for further analysis.

Upon the exploration of the nonlinear association of ACAG with CAP as well as CAS by the generalized additive model (GAM) and restricted cubic spline (RCS), respectively, the connection of ACAG to CAP and CSF was investigated using three generalized linear models (GLMs). The linear relationship between ACAG and NAFLD determined the use of logistic regression analysis to further analyze their relationship. When ACAG was coded into three groups according to the tertiles, its association with CAP, NAFLD, and CSF was also investigated and *P* for trend was calculated. Three models included crude model; age, waist circumference, diabetes, and hypertension adjusted model 1; age, waist circumference, diabetes, hypertension, HDL-C, triglycerides, glucose, AST, ALT, and BUN adjusted model 2. Stratified analysis according to waist circumference was performed to further analyze the association of ACAG as a continuous and categorical variable with NAFLD in normal and central obesity groups. Based on the European Society of Cardiology [17], waist circumference was divided into two categories: normal (Caucasian men < 94 cm; men of other ethnicities < 90 cm; women < 80 cm) and central obesity (Caucasian men ≥ 94 cm; men of other ethnicities ≥ 90 cm; women ≥ 80 cm).

Then, logistic regression and XG boost methods were used for ranking the feature importance and selecting the core variables for model construction. Receiver operating characteristic (ROC) curve analysis and decision curve analysis (DCA) were adopted to assess the clinical significance of the model based on ACAG. P < 0.05 indicated a significance level.

## Results

### Participant characteristics

Participant characteristics are summarized in Table 1. Participants in the NAFLD group especially in the NAFLD with CSF group tended to be older and had higher waist circumference (*P* < 0.05). A larger proportion of hypertension and diabetes individuals was observed with the severity of the disease (*P* < 0.05). Compared with the non-NAFLD group, levels of ACAG, ALT, AST, BUN, glucose, and triglyceride were significantly elevated in the NAFLD group, and exhibited an increased trend across NAFLD without CSF and NAFLD with CSF (*P* < 0.05). However, HDL-C level was significantly lower in the NAFLD group and exhibited a decreased trend across NAFLD without CSF and NAFLD with CSF.

**Table 1:** Baseline characteristics of participants in NHANES 2017–2018

	Overall	*P*	Non-NAFLD		NAFLD		*P* (Without CSF vs. with CSF
		(NAFLD vs. Non-NAFLD)		Total	Without CSF	With CSF	
	4531		3027	1504	1284	220	
Age	44.00 [24.00, 62.00]	< 0.001	37.00 [20.00, 60.00]	53.00 [37.00, 64.00]	52.00 [36.00, 64.00]	57.00 [43.00, 65.00]	0.006
Gender		< 0.001					0.306
Male	2089 (46.11)		1296 (42.82)	793 (52.73)	670 (52.18)	123 (55.91)	
Female	2442 (53.89)		1731 (57.18)	711 (47.27)	614 (47.82)	97 (44.09)	
Waist C (cm)	95.70 [83.80, 107.70]	< 0.001	89.40 [78.70, 99.80]	107.50 [98.50, 118.80]	106.00 [97.70, 116.80]	120.20 [110.20, 131.50]	< 0.001
PIR	2.16 [1.19, 4.08]	0.477	2.15 [1.18, 4.08]	2.20 [1.22, 4.08]	2.20 [1.22, 4.10]	2.19 [1.19, 3.88]	0.644
Smoking status		< 0.001					0.185
Never	3179 (70.16)		2231 (73.70)	948 (63.03)	811 (63.16)	137 (62.27)	
Former	807 (17.81)		454 (14.00)	353 (23.47)	293 (22.82)	60 (27.27)	
Current	545 (12.03)		342 (11.30)	203 (13.50)	180 (14.02)	23 (10.46)	
Hypertension		< 0.001					0.024
Yes	1355 (29.91)		681 (22.50)	674 (44.81)	560 (43.61)	114 (51.82)	
No	3176 (70.09)		2346 (77.50)	830 (55.19)	724 (56.39)	106 (48.18)	
Diabetes		< 0.001					< 0.001
Yes	552 (12.18)		224 (7.40)	328 (21.81)	244 (19.00)	84 (38.18)	
No	3979 (87.82)		2803 (92.60)	1176 (78.19)	104 (80.00)	136 (61.82)	
Total bilirubin (umol/L)	6.84 [5.13, 10.26]	0.074	6.840 [5.13, 10.26]	6.84 [5.13, 8.55]	6.84 [5.13, 8.55]	6.84 [5.13, 10.26]	0.003
Total calcium (mmol/L)	2.33 [2.28, 2.40]	< 0.001	2.33 [2.28, 2.40]	2.33 [2.28, 2.38]	2.33 [2.28, 2.38]	2.33 [2.25, 2.38]	0.308
ACAG (mmol/L)	18.45 [17.00, 19.90]	< 0.001	18.25 [16.85, 19.70]	18.80 [17.55, 20.25]	18.75 [17.50, 20.15]	19.05 [17.95, 20.95]	0.004
ALT (U/L)	17.00 [12.00, 24.00]	< 0.001	15.00 [11.00, 20.00]	22.00 [16.00, 32.00]	21.00 [15.00, 30.00]	27.00 [19.00, 43.00]	< 0.001
AST (U/L)	19.00 [16.00, 23.00]	< 0.001	18.00 [16.00, 22.00]	20.00 [17.00, 26.00]	20.00 [16.00, 25.00]	23.00 [19.00, 32.00]	< 0.001
BUN (mg/dL)	13.00 [11.00, 17.00]	< 0.001	13.00 [11.00, 16.00]	14.00 [11.00, 17.00]	14.00 [11.00, 17.00]	15.00 [12.00, 18.00]	0.038
Creatinine (mg/dL)	0.81 [0.67, 0.97]	< 0.001	0.80 [0.67, 0.95]	0.83 [0.69, 0.99]	0.83 [0.68, 0.98]	0.86 [0.69, 1.05]	0.102
Glucose (mg/dL)	92.00 [86.00, 100.00]	< 0.001	90.00 [85.00, 97.00]	97.00 [89.00, 111.00]	96.00 [89.00, 109.00]	101.00 [91.00, 127.00]	< 0.001
HDL cholesterol	51.00 [43.00, 61.00]	< 0.001	54.00 [46.00, 64.00]	46.00 [39.00, 54.00]	46.00 [40.00, 55.00]	43.00 [38.00, 51.00]	< 0.001
Triglyceride (mg/dL)	107.00 [75.00, 158.00]	< 0.001	93.00 [68.00, 134.00]	143.00 [102.00, 203.00]	141.00 [101.00, 201.00]	155.00 [113.00, 218.00]	0.044

*NAFLD* non-alcoholic fatty liver disease, *CSF* clinically significant fibrosis, *ACAG* albumin-corrected anion gap, *Waist C* waist circumference, *PIR* poverty income ratio, *ALT* alanine aminotransferase, *AST* aspartate aminotransferase, *BUN* blood urea nitrogen, *HDL* cholesterol, high-density lipoprotein cholesterol

### ACAG was associated with NAFLD but not the CSF

**Fig. 1:**
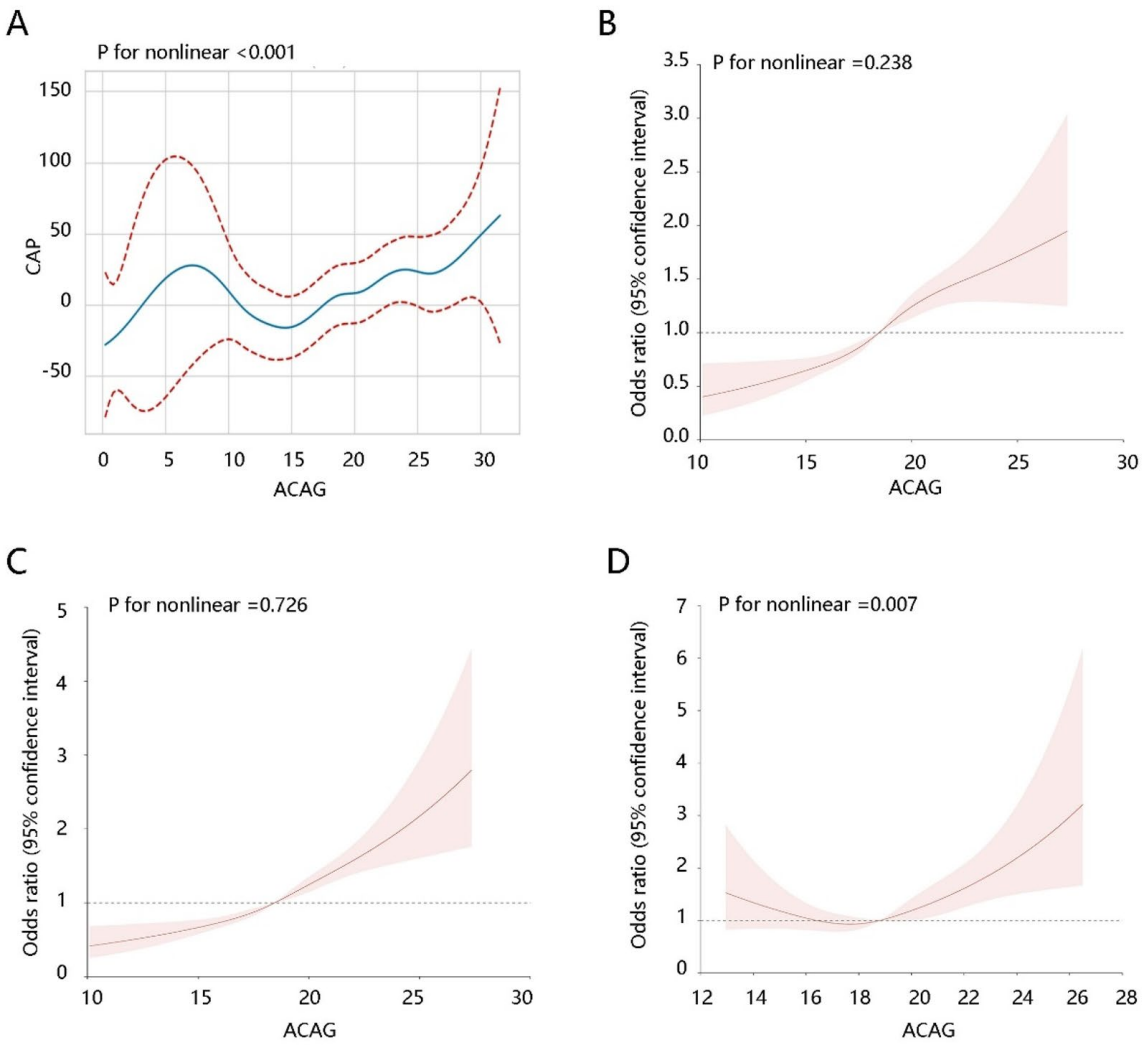
Generalized additive model and restricted cubic spline analyses. **A** Nonlinear relationship between albumin-corrected anion gap (ACAG) and controlled attenuation parameter (CAP). **B** Linear association of ACAG with non-alcoholic fatty liver disease (NAFLD) (CAP cutoff point: 285 dB/m). **C** Linear association of ACAG with NAFLD (CAP cutoff point: 263 dB/m). **D** Nonlinear relationship between ACAG and clinically significant fibrosis

First, we used GAM analysis to reveal the nonlinear relationship between ACAG and CAP (*P* for nonlinear < 0.001)** (**Fig. 1A**)**, and the GLM analysis was conducted to assess their relation further. There was no doubt that ACAG elevation was notably related to CAP in the crude model, and this significance still existed after adjusting various confounding factors in model 1 and model 2 (*P* < 0.05). Besides, the ACAG as a categorical variable was closely linked to CAP in three models (*P* < 0.05). As shown in Fig. 1B, C, ACAG was linearly related to NAFLD (P for nonlinear > 0.05). The positive correlations between ACAG level and NAFLD were consistent among the crude model, model 1, and model 2 (*P* < 0.05). An elevated ACAG concentration was positively related to NAFLD (OR, 1.421 [1.161–1.739]) and (OR, 1.286 [1.049–1.577]) in comparison with the first tertile ACAG in model 2. Similar results were observed when the CAP cutoff point was set up to 263 dB/m for the sensitivity analysis **(**Table 2**)**. *P* trend test confirmed that the elevated ACAG was connected with NAFLD (*P* for trend < 0.05). Of note, ACAG, age, waist circumference, HDL-C, triglycerides, glucose, BUN, and ALT were significantly related to both CAP and NAFLD (Additional file 1: Table S1).

**Table 2:** Association of ACAG with NAFLD

	β (95% CI)			OR (95% CI)			OR* (95% CI)		
Factors	Crude	Model 1	Model 2	Crude	Model 1	Model 2	Crude	Model 1	Model 2
ACAG level	3.746 (3.015, 4.478)	1.121 (0.475, 1.766)	1.166 (0.529, 1.802)	1.117 (1.088, 1.147)	1.047 (1.019, 1.081)	1.011 (1.005, 1.017)	1.130 (1.103, 1.159)	1.061 (1.029, 1.095)	1.064 (1.029, 1.101)
ACAG subgroups									
Tertile 1	Refs.	Refs.	Refs.	Refs.	Refs.	Refs.	Refs.	Refs.	Refs.
Tertile 2	13.389 (8.938, 17.841)	6.802 (2.959, 10.645)	5.232 (1.557, 8.907)	1.640 (1.402, 1.918)	1.539 (1.268, 1.867)	1.421 (1.161, 1.739)	1.486 (1.285, 1.718)	1.350 (1.124, 1.623)	1.247 (1.030, 1.510)
Tertile 3	22.353 (17.900, 26.807)	7.491 (3.561, 11.421)	5.814 (2.047, 9.580)	1.936 (1.658, 2.261)	1.387 (1.141, 1.685)	1.286 (1.049, 1.577)	1.986 (1.718, 2.296)	1.395 (1.157, 1.682)	1.323 (1.089, 1.607)
*P* for trend	< 0.001	< 0.001	0.002	< 0.001	0.001	0.017	< 0.001	< 0.001	0.004

Data in the table: *β*: CAP; OR: NAFLD (CAP cutoff point of 285 dB/m); OR*: NAFLD (CAP cutoff point of 263 dB/m).

*CAP* controlled attenuation parameter, *95% CI* 95% confidence interval, *OR* odds ratio, *ACAG* albumin-corrected anion gap

Moreover, the nonlinear relationship between the ACAG and CSF (*P* for nonlinear < 0.05) determined the use of GAM **(**Fig. 1D**)**. Although there was a significant association of ACAG with CSF in the crude mode, this relation was weakened after adjusting multiple covariates (*P* > 0.05) **(**Table 3**)**. The above findings indicated that ACAG might exhibit an essential role in predicting ACAG, but limited value for CSF prediction. Therefore, we focused on the association of ACAG with NAFLD in the following analyses. In addition to ACAG, age, waist circumference, HDL-C, triglycerides, glucose, BUN, and ALT might also play a certain role in NAFLD.

**Table 3:** Relationship between ACAG and CSF

	β (95% CI)		
Factors	Crude	Model 1	Model 2
ACAG level	0.010 (0.003, 0.018)	0.004 (− 0.003, 0.011)	0.002 (− 0.005, 0.010)
ACAG subgroups			
Tertile 1	Refs.	Refs.	Refs.
Tertile 2	0.035 (-0.009, 0.078)	0.013 (-0.031, 0.057)	0.010 (− 0.033, 0.053)
Tertile 3	0.053 (0.010, 0.097)	0.021 (− 0.023, 0.065)	0.015 (− 0.029, 0.058)
*P* for trend	0.016	0.343	0.509

*ACAG* albumin-corrected anion gap, *CSF* clinically significant fibrosis, *95% CI* 95% confidence interval

### Stratified analysis according to waist circumference

Moreover, we performed a stratified analysis between the ACAG and NAFLD in the normal and central obesity groups **(**Table 4**)**. In the normal group, ACAG had no obvious relation with CAP or NAFLD. There was a significant positive relation between ACAG and CAP both in the central obesity group. Subjects with middle or high tertile ACAG had 48.4% (OR, 1.484 [1.218, 1.807]) and 54.5% (OR, 1.545 [1.265, 1.888]) higher odds of NAFLD than those with low tertile ACAG in the central obesity group. These indicated that the association of ACAG with NAFLD varied in different waist circumference groups and mainly represented the role in the central obesity group.

**Table 4:** Association of ACAG with NAFLD according to waist circumference

	Normal	Central obesity
CAP	β (95% CI)	β (95% CI)
ACAG level	0.473 (-0.702, 1.648)	2.317 (1.506, 3.129)
ACAG subgroups		
Tertile 1	Refs.	Refs.
Tertile 2	3.72 (-2.739, 10.179)	9.485 (4.747, 14.223)
Tertile 3	1.524 (− 5.124, 8.171)	14.434 (9.625, 19.242)
*P* for trend	0.607	0.001
NAFLD (CAP cutoff point of 285 dB/m)	OR (95% CI)	OR (95% CI)
ACAG level	1.11 (0.966, 1.272)	1.069 (1.034, 1.107)
ACAG subgroups		
Tertile 1	Refs.	Refs.
Tertile 2	1.979 (0.876, 4.468)	1.484 (1.218, 1.807)
Tertile 3	1.984 (0.871, 4.522)	1.545 (1.265, 1.888)
*P* for trend	0.107	0.001

*ACA*G albumin corrected anion gap, *NAFLD* non-alcoholic fatty liver disease, *CAP* controlled attenuation parameter, *95% CI* 95% confidence interval, *OR* odds ratio

### Key variable selection and mediation analysis

**Fig. 2:**
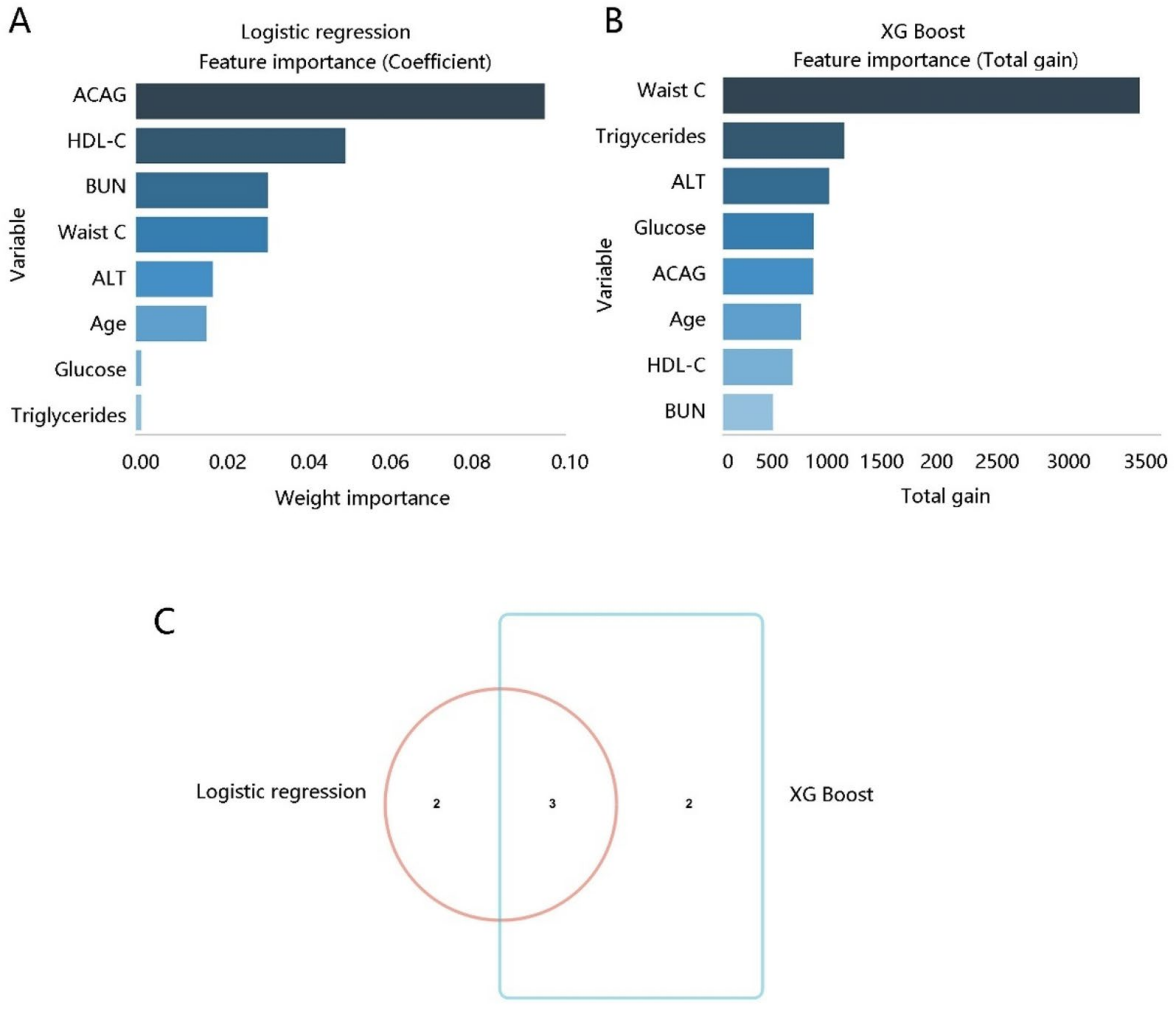
Key variable selection. Feature importance was ranked by **A** logistic regression, and **B** XG Boost method. **C** Venn analysis showed the intersection of the top five variables

Since the identification of several vital clinical factors significantly related to NAFLD, we utilized logistic regression and XG Boost methods to further select key variables by ranking feature importance** (**Fig. 2A, B**)**. The top five variables in each method were selected for the Venn analysis, and the results revealed three variables including ACAG, waist circumference, and ALT as the essential factors** (**Fig. 2C**)**, which drove our interest in investigating the mediating role of waist circumference and ALT in the relationship between ACAG and NAFLD. Expectedly, both waist circumference and ALT served as mediators in the association of ACAG with NAFLD (*P* < 0.001) **(**Table 5**)**.

**Table 5:** Mediating role of waist circumference and ALT in the relationship between ACAG and NAFLD

Path	Coefficients	*P*	Significance
Waist C ~ ACAG	1.050	< 0.001	Yes
NAFLD ~ waist C	0.014	< 0.001	Yes
Total effect	0.024	< 0.001	Yes
Direct effect	0.009	< 0.001	Yes
Indirect effect	0.014	< 0.001	Yes
ALT ~ ACAG	0.264	0.011	Yes
NAFLD ~ ALT	0.007	< 0.001	Yes
Total effect	0.024	< 0.001	Yes
Direct effect	0.022	< 0.001	Yes
Indirect effect	0.002	0.044	Yes

*Waist C* waist circumference, *ALT* alanine aminotransferase, *ACAG* albumin-corrected anion gap, *NAFLD* non-alcoholic fatty liver disease

### ACAG-based model construction and evaluation

**Fig. 3:**
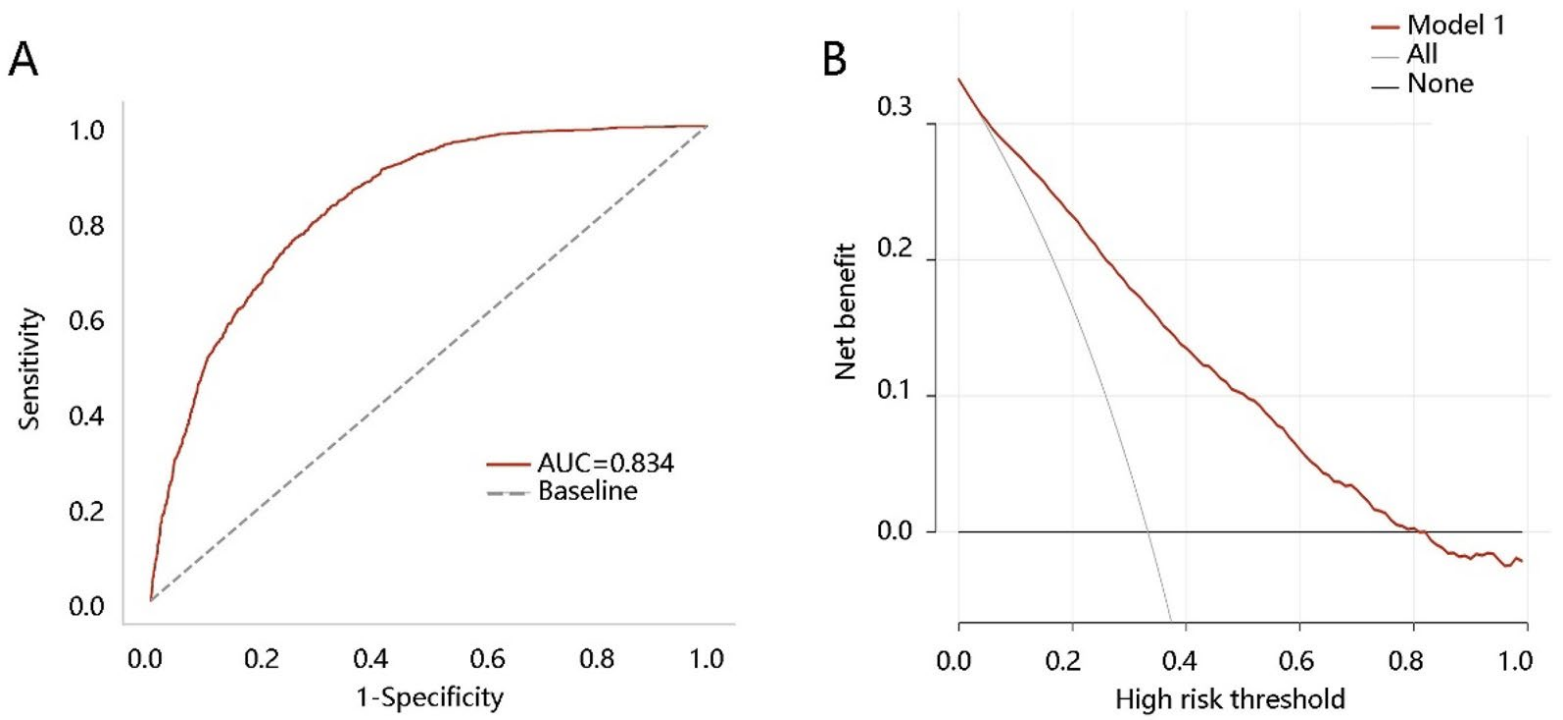
ACAG-based model evaluation. The clinical value of the ACAG-based model in predicting NAFLD by **A** receiver operating characteristic curve analysis and **B** decision curve analysis

To explore the clinical value of ACAG, we performed the ROC analysis and found that ACAG had a certain value for predicting NAFLD alone with an AUC of 0.578 (sensitivity, 0.766; specificity, 0.366) (Additional file 2: Table S2). Therefore, we conducted an ACAG-based model by integrating ACAG, waist circumference, and ALT using a logistic regression method. The risk score was calculated as the following formula: risk score = 0.028 * ALT + 0.076 * waist circumference + 0.047 * ACAG – 9.798. The ROC analysis result demonstrated that the area under the curve value of the ACAG-based model in predicting NAFLD was 0.834** (**Fig. 3A**)**. The DCA result showed the clinical efficacy of ACAG** (**Fig. 3B**)**. The above observations indicated that the ACAG-based model presented a satisfactory performance in predicting NAFLD.

## Discussion

In this study, we used data based on NHANES (2017–2018 cycles) and found that although ACAG elevation was significantly related to NAFLD and CSF, ACAG only served as an independent predictor for NAFLD.

NAFLD is one of the most prevalent liver disorders globally with an increasing incidence trend due to the epidemic of obesity and diabetes [18]. Traditionally, a biopsy is a gold standard for quantifying and diagnosing the fatty liver but with limited use in a broad population due to its invasive nature, elevated costs, and possible sampling error, which triggers the use of ultrasonography. Whereas, ultrasonography was only suitable and reliable when the degree of steatosis ≥ 20% [19]. The VCTE is a non-invasive and accurate method that is commonly adopted to examine the liver steatosis and fibrosis severity of NAFLD patients as well as the general population, and simultaneously record the CAP and LSM values [20]. This study employed VCTE and classified the participants according to CAP and LSM values with a cutoff point that exhibited high sensitivity and specificity. This reflects the scientific definition of NAFLD and CSF in this study. Besides, a high-quality diet, physical activity, and college education contributed to lower NAFLD risk using the NHANES data (2017–2018 cycles) [14]. The relationship between dietary-induced acid load and NAFLD has been investigated but with inconsistent results which may be explained by different study populations [3, 21]. Herein, this is the first large-scale study to investigate the association of ACAG with NAFLD and CSF.

After observing the highest ACAG level in the NAFLD with CSF group, followed by the NAFLD group and non-NAFLD group, we adopted three statistical models to observe the positive association of ACAG with NAFLD by correcting variables with different attributes, especially in the central obesity group. However, ACAG had no significant association with CSF. First, lipotoxicity plays a vital role in the NAFLD pathogenesis [22]. Lipotoxicity refers to the excessive production of cytosolic lipids (mainly triglycerides and free fatty acid subtypes) that adversely affect the metabolic pathways of the cell [23]. Accumulation of free fatty acids in the liver induces toxic metabolite formation, hence causing caspase-dependent apoptosis of endothelial cells, cardiomyocytes, and hepatocytes, leading to NAFLD [24, 25]. In addition to inducing apoptosis, the inflow of free fatty acids into hepatocytes also affects the functions of nuclear receptors and key enzymes associated with cholesterol metabolism, fatty acid oxidation, and hepatic de novo lipogenesis, thus further disrupting liver lipid metabolism. Second, glucotoxicity is a metabolic condition related to the increase in dietary sugar intake, which leads to hyperglycemia, inducing hepatotoxic effects by increasing steatosis [26]. Disturbance of metabolic flux increases the generation of harmful lipid intermediates followed by promoting reactive oxygen species (ROS) release and causing oxidative stress, facilitating NAFLD progression [27]. Moreover, ROS is involved in mediating endoplasmic reticulum stress, leading to the production of misfolded proteins, which is an essential factor in NAFLD [28]. Since mitochondria are critical in cellular oxygen consumption and ROS generation, the effect of lipotoxic on mitochondria may further aggravate oxidative stress [29]. Third, an essential pathophysiological process in NAFLD is the existence of liver inflammation, which can progress into hepatic fibrosis, and link the lipotoxic response with the oxidative stress generation [30]. Despite the important role of macrophages in inflammation response, hepatic stellate cells serve as the main driving factors of fibrosis response [31]. Collagens and related matrix proteins are secreted upon the activation of stellate cells, thereby generating fibrosis or scar tissue [32]. Therefore, it is reasonable to speculate that ACAG elevation might promote NAFLD via the involvement of lipotoxicity and glucotoxicity.

For strengths, a reliable and valid method FibroScan® was employed to measure hepatic steatosis and CSF. In addition, this is a large-scale population-based research, improving the evidence level of the results. Third, the variables we selected are cost-effective, and easy to get, contributing to the validation and execution of the results clinically. For limitations, this study is a cross-sectional study and ACAG cannot be considered as a risk factor, but rather as a marker related to NAFLD. Besides, the golden criterion of the definition of CSF in NAFLD is liver biopsy, and it has not reached a consensus on the definition of CSF in FibroScan, and hence it might misgroup according to FibroScan. Then, the underlying mechanism of the association of ACAG with NAFLD should be further explored by the experiments in the future.

In conclusion, the ACAG level is significantly higher in the NAFLD group than the non-NAFLD group, and the NAFLD with CSF group exhibits a higher ACAG level than the NAFLD without CSF group. ACAG might be a potential and robust biomarker for predicting NAFLD, especially in the overweight and obese group. Our study puts forward a valuable insight into the role of ACAG in NAFLD.

### Supplementary Information

**Additional file 1: Table S1.** The association of albumin-corrected anion gap with non-alcoholic fatty liver disease

**Additional file 2: Table S2.** The value of ACAG in predicting NAFLD.

## Data Availability

The data sets generated during and/or analyzed during the current study are available from the corresponding author on reasonable request.
