# Supplementary material for: Relationship between albumin-corrected anion gap and non-alcoholic fatty liver disease varied in different waist circumference groups: a cross-sectional study
Source: Eur J Med Res. 2024 Mar 27;29:203. doi: 10.1186/s40001-024-01811-w (PMC10967193; doi:10.1186/s40001-024-01811-w)
Supplement: Supplementary file 1 — Additional file 1: Table S1. The association of albumin-corrected anion gap with non-alcoholic fatty liver disease [file 40001_2024_1811_MOESM1_ESM.docx]

| **Table S1** The association of albumin-corrected anion gap with non-alcoholic fatty liver disease | | | |
| --- | --- | --- | --- |
| Factors | β (95% CI) | OR (95% CI) | OR* (95% CI) |
| ACAG | 1.166 (0.529, 1.802) | 1.046 (1.010, 1.083) | 1.064 (1.029, 1.101) |
| Age | 0.275 (0.176, 0.373) | 1.011 (1.005, 1.017) | 1.010 (1.005, 1.016) |
| Waist C | 1.705 (1.601, 1.809) | 1.067 (1.060, 1.073) | 1.070 (1.064, 1.077) |
| HDL-C | -0.252 (-0.373, -0.131) | 0.986 (0.979, 0.993) | 0.991 (0.984, 0.997) |
| Triglycerides | 0.077 (0.060, 0.094) | 1.004 (1.003, 1.005) | 1.004 (1.003, 1.005) |
| Glucose | 0.150 (0.095, 0.206) | 1.006 (1.003, 1.009) | 1.008 (1.004, 1.012) |
| BUN | -0.731 (-1.030, -0.433) | 0.979 (0.963, 0.994) | 0.981 (0.966, 0.996) |
| AST | -0.376 (-0.582, -0.169) | 0.985 (0.972, 0.998) | 0.990 (0.979, 1.001) |
| ALT | 0.723 (0.575, 0.872) | 1.032 (1.023, 1.042) | 1.027 (1.018, 1.036) |
| Diabetes | -4.155 (-9.508, 1.198) | 0.842 (0.645, 1.100) | 0.919 (0.692, 1.220) |
| Hypertension | -4.526 (-8.283, -0.769) | 0.805 (0.667, 0.972) | 0.868 (0.720, 1.048) |

**Data in the table:** β: CAP; OR: NAFLD (CAP cut-off point of 285 dB/m); OR*: NAFLD (CAP cut-off point of 263 dB/m). **Abbreviations:** CAP, controlled attenuation parameter; OR (95% CI), odds ratio (95% confidence interval); ACAG, albumin-corrected anion gap; waist C, waist circumference; HDL-C, high-density lipoprotein cholesterol; BUN, blood urea nitrogen; AST, Aspartate aminotransferase; ALT, Alanine aminotransferase.
