# Supplementary material for: Relationship between albumin-corrected anion gap and non-alcoholic fatty liver disease varied in different waist circumference groups: a cross-sectional study
Source: Eur J Med Res. 2024 Mar 27;29:203. doi: 10.1186/s40001-024-01811-w (PMC10967193; doi:10.1186/s40001-024-01811-w)
Supplement: Supplementary file 2 — Additional file 2: Table S2. The value of ACAG in predicting NAFLD. [file 40001_2024_1811_MOESM2_ESM.docx]

| **Table S2** The value of ACAG in predicting NAFLD | | | | | |
| --- | --- | --- | --- | --- | --- |
| Factor | AUC | Sensitivity | Specificity | Youden's index | Cutoff value |
| ACAG | 0.578 | 0.766 | 0.366 | 0.132 | 17.5 |

**Abbreviations:** ACAG, albumin-corrected anion gap; AUC, the area under the curve; NAFLD, non-alcoholic fatty liver disease.
